# TLR9 Rs352140 polymorphism contributes to a decreased risk of bacterial meningitis: evidence from a meta-analysis

**DOI:** 10.1017/S0950268820002666

**Published:** 2020-11-04

**Authors:** Haiyi Xue, Huan Peng, Jiaoming Li, Mingming Li, Song Lu

**Affiliations:** 1 Department of Critical Care Medicine, Sichuan Cancer Hospital & Institute, Chengdu 610041, People's Republic of China; 2 Department of Neurosurgery, Sichuan Cancer Hospital & Institute, Chengdu 610041, People's Republic of China; 3 Department of Neurosurgery, Chengdu Western Hospital, Chengdu 610036, People's Republic of China

**Keywords:** Bacterial meningitis, meta-analysis, polymorphism, Toll-like receptor 9

## Abstract

Some studies have suggested that the Toll-like receptor 9 polymorphism (TLR9 rs352140) is closely related to the risk of bacterial meningitis (BM), but this is subject to controversy. This study set out to estimate whether the TLR9 rs352140 polymorphism confers an increased risk of BM. Relevant literature databases were searched including PubMed, Embase, the Cochrane Library and China National Knowledge Infrastructure (CNKI) up to August 2020. Seven case-control studies from four publications were enrolled in the present meta-analysis. Odds ratios (OR) and confidence intervals (95% CI) were calculated to estimate associations between BM risk and the target polymorphism. Significant associations identified were allele contrast (A *vs.* G: OR 0.66, 95% CI 0.59–0.75, *P* = 0.000), homozygote comparison (AA *vs.* AG/GG: OR 0.62, 95% CI 0.49–0.78, *P* = 0.000), heterozygote comparison (A *vs.* G: OR 0.74, 95% CI 0.61–0.91, *P* = 0.005), recessive genetic model (AA *vs.* AG/GG: OR 0.78, 95% CI 0.65–0.93, *P* = 0.006) and dominant genetic model (AA *vs.* AG/GG: OR 0.70, 95% CI 0.57–0.85, *P* = 0.000). The findings indicate that, in contrast to some studies, the TLR9 rs352140 polymorphism is associated with a decreased risk for BM.

## Introduction

Bacterial meningitis (BM) is a common central nervous system infection associated with high mortality, and which has a significant negative impact on children's perception abilities [1, 2]. Early rapid diagnosis and timely treatment are key factors affecting its prognosis. At present, the diagnosis depends mainly on clinical characteristics and microbial and biochemical analysis of cerebrospinal fluid (CSF). However, non-specific clinical presentation and atypical changes in CSF composition can make it difficult to confirm the diagnosis of BM and distinguish it from viral meningitis and intracranial infection. As a consequence, there is an urgent need to identify reliable biomarkers for BM.

Various reports have demonstrated that pathogen recognizing receptors, especially Toll-like receptors (TLRs), play a vital role in modulating the host and immune responses to infection [3–6]. Besides activating immune responses, TLRs aid the elimination of bacteria and virus infections and play a key role in the innate immunity against microbial pathogens [7]. TLR9 has been suggested to regulate unmethylated cytosine-phosphate-guanine movement and influence the production of pro-inflammatory and anti-inflammatory cytokines [8, 9]. TLR9 gene polymorphisms have also been associated with multiple diseases such as pulmonary tuberculosis, duodenal ulcers and altered cytokines in gastric mucosa, systemic lupus erythematosus and chronic obstructive pulmonary disease [10–14]. Of these polymorphisms, the rs352140 variant has been the one most investigated, and some have suggested that it is significantly associated with susceptibility to BM [15], but this has not been supported by other studies [9, 16, 17]. We therefore set out in this study to establish through a meta-analysis of published data whether TLR9 rs352140 polymorphism confers an increased risk for BM.

## Materials and methods

### Search strategy

The databases PubMed, Embase, the Cochrane Library and the China National Knowledge Infrastructure were searched by applying the words: (‘TLR9’ or ‘Toll-like receptor 9’) together with (‘BM’ or ‘Bacterial meningitis’) up to the end of August 2020.

### Inclusion and exclusion criteria

The inclusion criteria met the following: (a) a case-control study; (b) reporting an assessment of association between TLR9 rs352140 polymorphism and BM risk; (c) offering sufficient information to allow the determination of OR and 95% CI values. Other publications such as letters and reviews were not included.

### Data extraction

Relevant information was extracted by the first and second authors and contradictory data or information was reassessed by the corresponding author. Ethical approval of our study was waived by the Ethics Committee of Sichuan Cancer Hospital＆Institute as no human or animal was directly enrolled in the study.

### Statistical analysis

The association power was assessed through the corresponding indexes including OR and 95% CI with the application of the Q- and *I*^2^ statistics [18, 19]. Four genetic models were applied including allele contrast (A *vs.* G), homozygote comparison (AA *vs.* GG), recessive genetic model (AA *vs.* GA/GG) and dominant genetic model (AA/GA *vs.* GG). The models of fixed-effects and random-effects were utilised to assess the degree of heterogeneity [20, 21] while Funnel plots and Egger's test were used to identify publication bias [22]. The Hardy–Weinberg Equilibrium (HWE) which states that ‘genotype frequencies in a population remain constant between generations in the absence of disturbance by outside factors’ [23] was used to assess the validity of the published series; only studies with control groups having a *P* value for the HWE > 0.05 were included. All statistical analyses were performed using Stata 12.0 (Stata Corporation, Los Angeles, USA).

## Results

### Meta-analysis studies

**Fig. 1.**
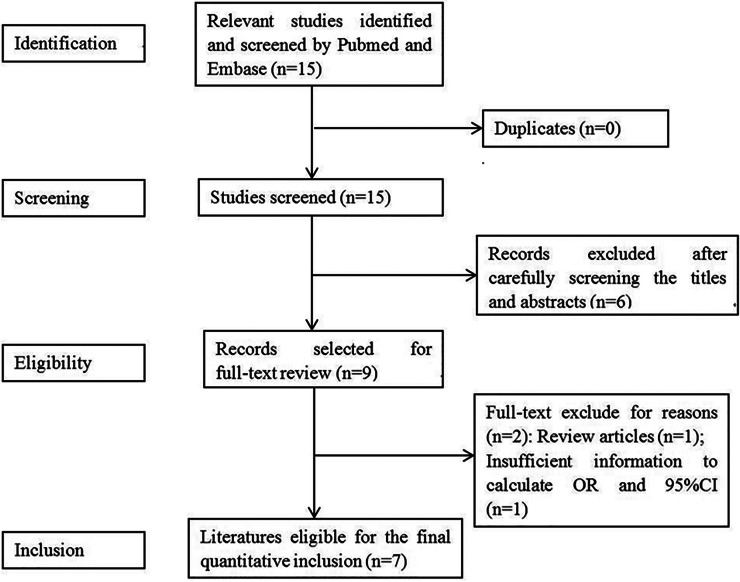
Flow diagram for identification of eligible studies for this meta-analysis.

Figure 1 shows the flow chart of meta-analysis search course. Based on the search strategy, four studies reporting seven groups of patients – three comprising both meningococcal (MM) and pneumococcal (PM) cases [9, 16, 17], and one reporting BM cases as a whole were identified [15] (Table 1). All but Wang's study used population-based controls.

**Table 1.** Main characteristics of the case-control studies included in meta-analysis

Literature	Country	Sample size	Studied type of BM	HWE (*P* value)
Gowin *et al.* [17]	Poland	25/49	NM, PM	0.812
Wang *et al.* [15]	China	126/252	NM	0.940
van Well *et al.* [16]	The Netherlands	327/392	NM, PM	0.843
Sanders *et al.* [9]	The Netherlands	380/392	NM, PM	0.843

HWE, Hardy–Weinberg equilibrium; PM, pneumococcal meningitis; MM, meningococcal meningitis.

### Meta-analysis results

The results of the meta-analysis of the association of TLR9 rs352140 polymorphism and BM susceptibility are shown in Table 2 and Supplementary Material. Positive associations were found by allele contrast (A *vs.* G: OR 0.66, 95% CI 0.59–0.75, *P* = 0.000), homozygote comparison (AA *vs.* AG/GG: OR 0.62, 95% CI 0.49–0.78, *P* = 0.000), recessive genetic model (AA *vs.* AG/GG: OR 0.78, 95% CI 0.65–0.93, *P* = 0.006) and dominant genetic model (AA *vs.* AG/GG: OR 0.70, 95% CI 0.57–0.85, *P* = 0.000)

**Table 2.** Meta-analysis of the TLR9 rs352140 polymorphism with BM risk

Comparison	Group of BM	*N*	Test of association			Mode	Test of heterogeneity		
			OR	95% CI	*P*		*χ*^2^	*P*	*I*^2^
A *vs*. G	Overall	7	0.66	0.59–0.75	0	Fixed	6.45	0.374	7.0
	Caucasian	6	0.65	0.57–0.74	0	Fixed	6.23	0.284	19.8
	Asian	1	0.71	0.52–0.97	0.034	Fixed	0	–	–
	PM	3	0.80o	0.62–1.02	0.073	Fixed	1.29	0.525	0
	MM	3	0.61	0.52–0.71	0	Fixed	1.68	0.432	0
	Mixed	1	0.71	0.52–0.97	0.034	Fixed	0	–	–
AA *vs*. GG	Overall	7	0.62	0.49–0.78	0	Fixed	5.22	0.516	0
	Caucasian	6	0.65	0.51–0.83	0.001	Fixed	4.24	0.516	0
	Asian	1	0.43	0.21–0.92	0.028	Fixed	0	–	–
	PM	3	0.92	0.57–1.50	0.742	Fixed	0.48	0.788	0
	MM	3	0.57	0.43–0.76	0	Fixed	1.07	0.585	0
	Mixed	1	0.43	0.21–0.92	0.028	Fixed	0	–	–
GA *vs*. GG	Overall	7	0.74	0.61–0.91	0.005	Fixed	2.86	0.826	0
	Caucasian	6	0.73	0.58–0.92	0.008	Fixed	2.75	0.738	0
	Asian	1	0.80	0.50–1.26	0.327	Fixed	0	–	–
	PM	3	0.82	0.52–1.31	0.414	Fixed	0.33	0.849	0
	MM	3	0.71	0.54–0.92	0.010	Fixed	2.10	0.350	4.7
	Mixed	1	0.80	0.50–1.26	0.327	Fixed	0	–	–
AA *vs*. GA/GG	Overall	7	0.78	0.65–0.93	0.006	Fixed	5.67	0.461	0
	Caucasian	6	0.81	0.67–0.97	0.024	Fixed	3.87	0.568	0
	Asian	1	0.49	0.24–0.99	0.048	Fixed	0	–	–
	PM	3	1.07	0.75–1.52	0.724	Fixed	0.37	0.830	0
	MM	3	0.73	0.59–0.91	0.004	Fixed	0.30	0.862	0
	Mixed	1	0.49	0.24–0.99	0.048	Fixed	0	–	–
AA/GA *vs*.GG	Overall	7	0.70	0.57–0.85	0	Fixed	3.38	0.760	0
	Caucasian	6	0.70	0.56–0.86	0.001	Fixed	3.38	0.642	0
	Asian	1	0.70	0.46–1.09	0.116	Fixed	0	–	–
	PM	3	0.86	0.56–1.34	0.514	Fixed	0.44	0.803	0
	MM	3	0.65	0.51–0.83	0.001	Fixed	1.71	0.426	0
	Mixed	1	0.70	0.46–1.09	0.116	Fixed	0	–	–

PM, pneumococcal meningitis; MM, meningococcal meningitis.

No significant heterogeneity was found in any genetic model (*P* > 0.1 and *I*^2^ < 50%) and the results of each of the single studies did not alter the key associations shown by the meta-analysis. We did not find any obvious asymmetry in the funnel plot (*P* = 0.107) (figure not shown), and there was also no evident publication bias by Egger's test in the recessive genetic model (*P* = 0.194).

## Discussion

Although several pathogenic microorganisms may cause infection of the meninges, relatively few bacterial species, namely – *Streptococcus pneumoniae*, *Neisseria meningitidis*, *Escherichia coli* and *Listeria monocytogenes* are associated with the majority of cases [24]. BM is a common acute infection of the central nervous system in children. In developed countries, its incidence is reported to range from 1.4 to 6/100 000, with a fatality rate of approximately 5% [25]. The incidence rate in developing countries is more than 10 times higher and accompanied by a higher fatality rate [26]. Moreover, 10–20% of survivors have some neurological sequelae including cognitive or visual impairment, bilateral hearing loss, movement disorders and epilepsy which impact significantly on patients' life conditions. It is widely recognised that several factors contribute to susceptibility to BM. Subjects most at risk of high mortality and morbidity are the newborn, particularly in poor countries, and exposed to bacterial infections, especially due to Gram-negative bacilli and *S. pneumoniae* [27–29]. Apart from the impact of demographic factors such as age, environment and living conditions, genetic predisposition to BM has been considered as a significant contributor to the disease occurrence. The relationship between the TLR9 rs352140 polymorphism and susceptibility to BM has been investigated since 2011 [9] but no firm conclusion regarding such an association has emerged. A meta-analysis of relevant published studies was therefore warranted, and to the best of our knowledge, this investigation is the first to study the specific relationship between TLR9 rs352140 polymorphism and BM susceptibility. The analysis indicated that this polymorphism is more associated with a decreased risk to BM. We consider that such a conclusion is reliable according to the heterogeneity test, sensitivity analysis and publication bias matrices. Furthermore, each of the studies analysed satisfied the HWE which states that allele and genotype frequencies in a defined population will remain constant from generation to generation in the absence of other evolutionary influences.

This study has some limitations. Notably, the total sample size could be considered to be insufficient owing to the lack of relevant well-designed original studies with a greater number of patients; such studies remain urgently needed. Secondly, a wider range of ethnicities are warranted to give a greater variety of racial and genetic backgrounds. Lastly, the four studies analysed did not take into consideration confounding factors such as age, gender and radiation exposure which might have contributed to some bias in our results.

In conclusion, our meta-analysis of the available studies in the literature indicates that, in contrast to some studies, the TLR9 rs352140 polymorphism is associated with a decreased risk for BM.

## Supplementary material

For supplementary material accompanying this paper visit https://doi.org/10.1017/S0950268820002666. click here to view supplementary material

## Availability of data and materials

The datasets used and/or analysed during the present study are available from the corresponding author on reasonable request.
