# Supplementary figures and images for: TLR9 Rs352140 polymorphism contributes to a decreased risk of bacterial meningitis: evidence from a meta-analysis
Source: Epidemiol Infect. 2020 Nov 4;148:e294. doi: 10.1017/S0950268820002666 (PMC7770465; doi:10.1017/S0950268820002666)

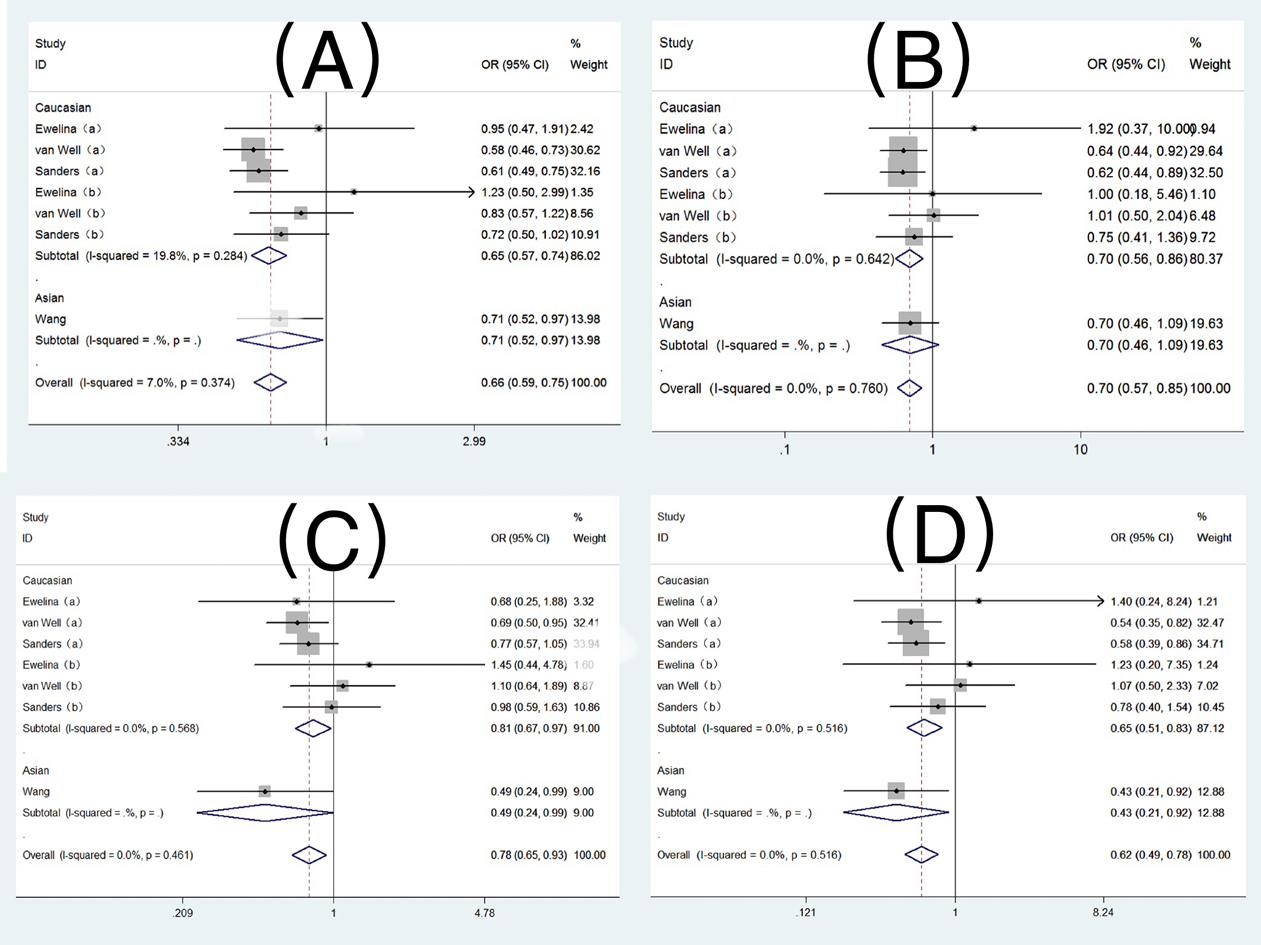

Supplement: Supplementary file 1 [file S0950268820002666sup001.tif]
